# Interaction of CD14 haplotypes and soluble CD14 on pulmonary function in agricultural workers

**DOI:** 10.1186/s12931-017-0532-y

**Published:** 2017-03-16

**Authors:** Tricia D. LeVan, Lynette M. Smith, Art J. Heires, Ted R. Mikuls, Jane L. Meza, Lisa A. Weissenburger-Moser, Debra J. Romberger

**Affiliations:** 1 Department of Epidemiology, University of Nebraska Medical Center, 985910, Omaha, NE 68198-5910 USA; 2 Department of Internal Medicine and Veterans Nebraska Western Iowa Healthcare System, Omaha, NE USA; 3 Department of Biostatistics, University of Nebraska Medical Center, Omaha, NE USA; 4 Department of Internal Medicine, University of Nebraska Medical Center, Omaha, NE USA

**Keywords:** CD14, Agriculture, Lung function, COPD, Polymorphism

## Abstract

**Background:**

Agricultural environments are contaminated with organic dusts containing bacterial components. Chronic inhalation of organic dusts is implicated in respiratory diseases. CD14 is a critical receptor for gram-negative lipopolysaccharide; however, its association with respiratory disease among agricultural workers is unknown. The objective of this study was to determine if serum soluble CD14 (sCD14) levels are associated with lung function among agricultural workers and if this association is modified by genetic variants in *CD14*.

**Methods:**

This cross-sectional study included *584* veterans with >2 years of farming experience and that were between the ages of 40 and 80 years. Participants underwent spirometry and were genotyped for four tagging *CD14* polymorphisms *(CD14*/-2838, rs2569193; *CD14*/-1720, rs2915863; *CD14*/-651, rs5744455; and *CD14*/-260, rs2569190). Serum sCD14 was assayed by ELISA.

**Results:**

Subjects were 98% white males with a mean age 64.5 years. High soluble CD14 levels (> median sCD14) were associated decreased lung function (FEV_1_/FVC, *p* = 0.011; % predicted FEV_1_, *p* = 0.03). When stratified by COPD (yes/no) and smoking status (ever/never), high sCD14 levels (> median sCD14) were associated with low lung function among ever smokers with COPD (% predicted FEV_1_, p_adj_ = 0.0008; FEV_1_/FVC, p_adj_ = 0.0002). A similar trend was observed for never smokers with COPD; however, results did not reach statistical significance due to small sample size. There was a significant sCD14 x COPD/smoking interaction with lung function (% predicted FEV_1_, p_inter_ = 0.0498; FEV_1_/FVC, p_inter_ = 0.011). Regression models were adjusted for age, body mass index, education, sex, race and years worked on a farm. No association was found between *CD14* polymorphisms/haplotypes (*CD14*/-2838; *CD14*/-1720*; CD14*/-651; *CD14*/-260) and sCD14 levels. The final model included the variables sCD14 and haplotypes and a haplotype x sCD14 interaction term. Individuals with the GTTG haplotype (*CD14*/-2838 → *CD14*/-260) and high sCD14 levels (> median sCD14) had on average 6.94 lower % predicted FEV_1_ than individuals with the GCCA haplotype and low sCD14 levels (≤ median sCD14, p_adj_ = 0.03).

**Conclusion:**

*CD14* haplotypes and sCD14 are important mediators of lung function among those with COPD in this occupationally-exposed population.

**Electronic supplementary material:**

The online version of this article (doi:10.1186/s12931-017-0532-y) contains supplementary material, which is available to authorized users.

## Background

CD14 is a pattern-recognition receptor and exists as two distinct forms: as a glycosyl-phosphatidylinositol (GPI) - anchored membrane protein on the surface of monocytes, macrophages and neutrophils and as a monocyte or liver-derived serum soluble protein (sCD14) lacking the GPI anchor [1]. Soluble CD14 is an acute phase protein [2], but is also found in normal serum at microgram concentrations [3], and confers sensitivity to a gram-negative bacterial cell wall component, i.e. lipopolysaccharide (LPS), for cells lacking membrane CD14, such as endothelial and epithelial cells [4]. Together with LPS and LPS-binding protein, CD14 forms a ligand that interacts with the toll-like receptor – 4 (TLR-4)/MD-2 receptor complex and leads to activation of innate host defense mechanisms, stimulating numerous Th_1_ proinflammatory cytokines, including tumor necrosis factor-α (TNF-α) and interleukin-6 (IL-6) [5]. Recently, it has been suggested that CD14 also can interact with other pathogen-associated molecular patterns (PAMPs) such as acylated lipoproteins and peptidioglycan from gram-positive bacteria, and can participate in the formation of multi-receptor complexes other than TLR-4, e.g. TLR-1, -2 and -6 [6].

Farming environments are highly contaminated with airborne inhalable organic dust [7, 8], which contains PAMPs, including gram-negative and gram-positive bacterial components [9, 10]. Forty % of these dust particles are in the respirable range with a median diameter of 4 μm or less, which may be deposited at the level of the terminal bronchioles and alveoli [7, 11]. Chronic inhalation of complex organic dust is implicated in respiratory disease development and severity, including rhinitis, sinusitis, asthma-like syndrome, organic dust toxic syndrome, chronic bronchitis, and chronic obstructive pulmonary disease (COPD) [12]. Several studies associate LPS levels in the agricultural environment with adverse respiratory health outcomes. In healthy individuals, inhalation of purified LPS induces dose-related symptoms, a decrease in lung function and diffusion capacity, airway obstruction and both bronchial and systemic inflammation [13–17].

While these studies suggest an important role of LPS in respiratory disease pathogenesis, the function of sCD14, a critical receptor for LPS, in host defense and respiratory disease among agricultural workers has not been defined. In fact, no large-scale comprehensive studies have examined the relationship of sCD14 concentrations with measures of lung function in occupationally- or non-occupationally- exposed individuals. The limited numbers of small studies in non-occupationally exposed adults have shown that sCD14 levels are elevated in ever smokers and COPD patients (LPS is a component of cigarette smoke), and humans experimentally exposed to LPS compared to healthy non-smokers [14, 18].

Soluble CD14 levels also have been shown to be influenced by *CD14* polymorphisms in diverse populations such as infants, patients with cardiovascular disease, tuberculosis, periodontal disease and healthy persons [14, 19–23]. Whether the association between sCD14 levels and lung function is modified by *CD14* polymorphisms has not been investigated.

As part of the present study, we utilized cross-sectional data from a well-characterized population of agricultural workers from the Midwest to determine if sCD14 concentration was associated with lung function. We hypothesized that circulating concentrations of sCD14 are elevated in agricultural workers with impaired lung function compared to those with higher lung function. We also examined whether well-characterized polymorphisms and haplotypes in the *CD14* gene modify this association.

## Methods

### Study population and clinical assessments

This is a cross-sectional study of U.S veterans with agricultural exposure recruited from the outpatient clinics at the Omaha Veterans Affairs Medical Center. During a clinic visit, the veteran was asked the following question, “Have you worked on a farm for more than two years?”

Those who answered “yes” to this question and were between the ages of 40 and 80 years of age were eligible for the study. Based on self-report and medical chart confirmation, participants had no history of asthma, lung cancer, metastatic cancer to the lungs or interstitial lung diseases such pulmonary fibrosis, sarcoidosis or hypersensitivity pneumonitis. Atopy was not assessed in the participants. Those with a history of an infection or exacerbation within the previous three weeks were excluded from the study. Recruitment into the study began March 2008 and continued through December 2013, with a total 681 participants. Demographic information, smoking status and years worked on a farm were obtained by self-report at the time of enrollment. Smokers were defined as having smoked more than 100 cigarettes in their lifetime [24]. Blood was obtained by venipuncture and used for cell differentials, serum sCD14 and genomic DNA isolation. All participants underwent spirometry with post-bronchodilator spirometry (0.083% albuterol) performed on veterans with a FEV_1_/FVC < 0.70. COPD was defined by the Global Initiative for Chronic Obstructive Lung Disease (GOLD) classification criteria as FEV_1_/FVC < 0.70 [25]. The highest recorded FEV_1_ and FVC were used to derive height-, weight-, age-, gender- and ethnic- adjusted values based on National Health and Nutrition Survey (NHANESIII) reference equations [26]. The study was approved by the VA Institutional Review Board, and all participants signed a written informed consent document before enrollment.

### Soluble CD14 ELISA

Soluble CD14 in serum was quantified using a commercially available kit (DuoSet ELISA development system, R & D Systems, Inc., Minneapolis, MN, USA) (Supplement Methods). The limit of detectability was 125 pg/ml.

### Genetic analysis

The complete coding region of *CD14*, intronic sequence, 6 kb of 5’ genomic and 2 kb of 3’ genomic DNA were analyzed, and tagging single nucleotide polymorphisms (SNPs) were chosen based on a minor allele frequency > 5% and linkage disequilibrium (LD) <0.8 [27]. Additional SNPs were included based on their functional significance and relevant citations in the literature. The following SNPs were analyzed for this study, nomenclature relative to translation start site: *CD14*/-2838, rs2569193; *CD14*/-1720, rs2915863; *CD14*/-651, rs5744455; and *CD14*/-260, rs2569190. Aliases relative to the transcription start site are *CD14*/-2737, *CD14*/-1619, *CD14*/-550, and *CD14*/-159, respectively. Genomic DNA was isolated from whole blood using the QiaAMP DNA Blood and Tissue Mini Kit (Qiagen, Valencia, CA, USA). DNA samples were genotyped using matrix-assisted laser desorption/ionization time-of-flight mass spectrometry (MALDI-TOF; Agena Bioscience, San Diego, CA, USA). Multiplex polymerase chain reaction assays and associated extension reactions were designed using SpectroDesigner software (Agena Bioscience). Primer extension products were loaded onto a 384-element chip with a nanoliter pipetting system (Agena Bioscience) and analyzed with a MassArray mass spectrometer (Bruker Daltonik GmbH, Bremen, Germany). The resulting mass spectra were analyzed for peak identification using SpectroTyperRT 4.0 software. For genotyping quality control, Hardy-Weinberg calculations were performed to ensure that each marker was within the expected allelic population equilibrium.

### Statistical analyses

Continuous lung function variables (% predicted FEV_1_ and FEV_1_/FVC ratio) were compared by patient characteristics using *t*-test and ANOVA. Soluble CD14 levels were categorized at the median value (≤ median, > median) due to right-handed skew and bimodal distribution; therefore, a transformation would not be able to normalize the data. Because of the bimodal distribution, we felt it most appropriate to categorize soluble CD14 at the median. Associations with patient characteristics and lung function variables were assessed using chi-square and t-tests. Because smoking history and COPD are directly related to lung function, a combination variable (COPDsmoke) was made to assess the interaction between sCD14 and lung function. COPDsmoke contained 4 categories: 1) COPD, ever smoker, 2) COPD, never smoker, 3) no COPD, ever smoker, and 4) no COPD, never smoker. The effect of sCD14 (≤ median, > median) and COPDsmoke on lung function was examined in 2-way ANOVA models, with the lung function variable as the outcome, fixed effects for sCD14 (≤ median, > median) and COPDsmoke, and the sCD14 (≤ median, > median) x COPDsmoke interaction term included in the model. Multivariable linear regression models were examined considering the sCD14 (≤ median, > median) x COPDsmoke interaction on lung function, while adjusting for age, body mass index (BMI), education, sex, race and years worked on a farm. P-values for pairwise comparisons were adjusted with Tukey’s method, a standard technique that considers all possible pairwise differences of means at the same time.

Associations between sCD14 (≤ median, > median) and *CD14* polymorphisms were assessed using chi-square tests. The effect of *CD14* polymorphisms on lung function was assessed with univariate ANOVA and multivariable linear regression. Multiplicative interactions of *CD14* polymorphisms x sCD14 (≤ median, > median) or *CD14* polymorphisms x COPDsmoke on lung function were tested, and neither were found to be statistically significant. Multivariable models were adjusted for age, BMI, education, sex, COPD status, race and years worked on a farm.

*CD14* haplotypes were constructed using Haploview software, and haplotype blocks were estimated using the confidence interval for R^2^ values. Haplotypes were defined as *CD14*/-2838, *CD14*/-1720, *CD14*/-651 and *CD14*/-260. The association between sCD14 and *CD14* haplotypes was tested using the R function haplo.score. The effect of a multiplicative interaction of sCD14 (≤ median, > median) x *CD14* haplotypes on lung function was also evaluated in regression models, assuming an additive model for the haplotypes. The interaction between COPDsmoke x CD14 haplotypes was not significant and removed from the models. Modelling the effect of haplotypes was conducted with the R package haplo.stats and the haplo.glm and haplo.score functions. Haplo.glm fits multivariable linear regressions of lung function on haplotype, allowing for ambiguous haplotypes, interactions and covariates. This method performs an iterative two-step expectation-maximization (EM) algorithm, with the posterior probabilities as weights to update the regression coefficients, and the regression coefficients are used to update the posterior probabilities . Models that do not include haplotype information were fit using SAS 9.3 (SAS Institute Inc., Cary, NC, USA).

## Results

### Subject characteristics

Study population characteristics stratified by lung function (% predicted FEV_1_ and % FEV_1_/FVC) are summarized in Table 1. There were a total of 681 veterans in the population; however, only 584 patients had complete data on pulmonary function measures and covariates. Those with missing data were excluded in the analysis. Individuals with missing data had similar demographics (age, BMI, education, sex, race, smoking status, COPD and mean years worked on a farm) compared to those included in the analysis (data not shown). Reflecting demographic trends of the VA population in the urban Midwest [28], patients were predominately white males (98%) with a mean age of 64.8 years. The population included 230 patients with COPD, as defined by FEV_1_/FVC < 0.70, and 354 without COPD. Individuals with low pulmonary function values (% FEV_1_ and % FEV_1_/FVC) were more likely to be older, have lower BMI, have less education, and be ever smokers. Lung function values were independent of years worked on a farm, exposure to crops, and exposure to animals.

**Table 1:** Characteristics of study population by lung function

	Total N	FEV_1_ (% predicted) Mean (SD)	FEV_1_/FVC (%) Mean (SD)
Age, years			
39-50	38	87.6 (11.8)	74.2 (7.1)
51-60	117	80.8 (18.8)	71.4 (10.9)
61-70	274	80.2 (21.6)	70.3 (11.7)
71-80	155	77.0 (23.3)	66.0 (12.2)
Sex			
Male	571	79.6 (21.2)	69.4 (11.7)
Female	13	92.3 (14.1)	77.3 (6.1)
BMI			
< 25	86	72.4 (25.9)	61.8 (14.9)
25-29.9	171	81.3 (22.2)	68.5 (11.7)
≥ 30	327	81.2 (18.8)	72.3 (9.5)
Race			
White	555	79.8 (21.1)	69.6 (11.7)
Other	23	87.7 (20.9)	71.8 (9.0)
Education			
≤ High School	250	77.0 (22.3)	67.3 (12.6)
> High School	311	82.8 (19.6)	71.4 (10.3)
Smoking Status			
Ever	463	77.9 (21.4)	68.3 (12.1)
Never	113	88.5 (16.6)	75.2 (6.6)
COPD			
No	354	88.6 (16.6)	76.6 (4.3)
Yes	230	66.6 (20.5)	58.9 (11.3)
Steroid Use (Inhaled, Oral)^a^			
No	507	83.4 (18.9)	71.6 (9.7)
Yes	77	56.6 (20.8)	57.0 (15.2
Worked on a Farm, years			
< 10	93	76.7 (20.9)	69.5 (11.8)
10-19.9	158	83.1 (18.8)	70.7 (10.3)
20-29.9	92	79.0 (21.5)	68.9 (12.1)
30+	228	79.5 (22.5)	69.3 (12.2)
Exposure to crops			
No	70	77.4 (22.4)	68.9 (12.0)
Yes	499	80.4 (20.9)	69.8 (11.5)
Exposure to animals			
No	63	77.0 (22.2)	70.4 (12.0)
Yes	506	80.4 (21.0)	69.6 (11.5)

*Abbreviations and Definitions: BMI* body mass index (kg/m^2^), *FEV*_*1*_ forced expiratory volume in 1 s, *% FEV*_*1*_ */FVC* FEV_1_/FVC x 100, *FVC* forced vital capacity, *COPD* FEV_1_/FVC < 0.70

^a^In the last 12 months have you taken inhaled steroids (yes/no), Advair (yes/no) or Prednisone (yes/no)?

### Risk factors associated with sCD14 concentration

Soluble CD14 levels were categorized as ≤ median or > median. Age and BMI were determinants of sCD14 levels (Table 2). The mean age for participants with sCD14 levels > median was greater (66.0 years) compared to those with sCD14 levels ≤ median (63.6 years) (*p* = 0.0057). Individuals with BMI less than 30 kg/m^2^ were more likely to have sCD14 levels > median than those with a BMI ≥ 30 kg/m^2^ (*p* = 0.01). Surprisingly, none of the farming variables (years worked on a farm, exposure to crops, exposure to animals) were statistically associated with sCD14 levels.

**Table 2:** Risk Factors Associated with Serum sCD14 Concentration

	sCD14 ≤ median (*n* = 292) *N* (%)	sCD14 > median (*n* = 292) *N* (%)	*p*-value
Age, years			
39-50	22 (8%)	16 (5%)	0.0057
51-60	70 (24%)	47 (16%)	
61-70	139 (48%)	135 (46%)	
71-80	61 (21%)	94 (32%)	
Sex			
Male	283 (97%)	288 (99%)	0.16
Female	9 (3%)	4 (1%)	
BMI			
< 25	34 (12%)	52 (18%)	0.01
25-29.9	77 (26%)	94 (32%)	
≥ 30	181 (62%)	146 (50%)	
Race			
White	275 (95%)	280 (97%)	0.29
Other	14 (5%)	9 (3%)	
Education			
≤ High School	118 (42%)	132 (48%)	0.15
> High School	166 (58%)	145 (52%)	
Smoking Status			
Ever	225 (78%)	238 (83%)	0.13
Never	64 (22%)	49 (17%)	
COPD			
No	181 (62%)	173 (59%)	0.50
Yes	111 (38%)	119 (41%)	
Steroid Use (Inhaled, Oral)^a^			
No	260 (89%)	247 (85%)	0.11
Yes	32 (11%)	45 (15%)	
FEV_1_/FVC (mean ± SD)	70.9 ± 10.1	68.4 ± 13.0	0.011
FEV_1_ (%) (mean ± SD)	81.8 ± 19.4	78.0 ± 22.6	0.030
Worked on a Farm, years			
< 10	48 (17%)	45 (16%)	0.23
10-19.9	81 (28%)	77 (27%)	
20-29.9	54 (19%)	38 (13%)	
30+	105 (36%)	123 (43%)	
Exposure to crops			
No	31 (11%)	39 (14%)	0.26
Yes	257 (89%)	242 (86%)	
Exposure to animals			
No	25 (9%)	38 (14%)	0.06
Yes	263 (91%)	243 (86%)	

*Abbreviations and Definitions: BMI* body mass index (kg/m^2^), *COPD*, FEV_1_/FVC < 0.70

^a^In the last 12 months have you taken inhaled steroids (yes/no), Advair (yes/no) or Prednisone (yes/no)?

### Association of sCD14 levels with lung function

**Fig. 1:**
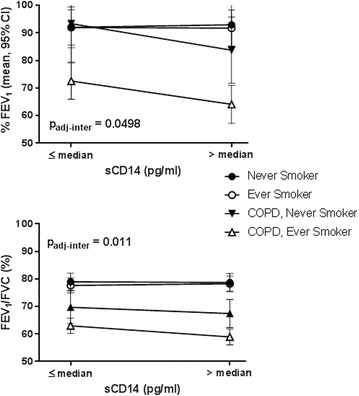
Interaction of sCD14 and COPD on lung function. X-axis represents ≤ or > median levels of sCD14. Y-axis represents lung function (adjusted means and 95% confidence interval). Top. % predicted FEV_1_: interaction, p_adj-inter_ = 0.0498, Bottom. % FEV_1_/FVC: interaction p_adj-inter_ = 0.011. Interaction results are adjusted for age, education, sex, race, BMI and years worked on a farm

Because impaired lung function has been shown to be associated with systemic inflammation and CD14 is a pivotal receptor for PAMPs found in agricultural dusts, we investigated the association between serum sCD14 levels and pulmonary function among agricultural workers. In univariate models we found that there was an association between sCD14 concentration and the lung function variables FEV_1_ (% predicted, *p* = 0.03) and FEV_1_/FVC (%, 0.011) (Table 2). In regression models we included a sCD14 x COPDsmoke multiplicative interaction term because COPD and smoking status are related to lung function. We found statistically significant interactions with % predicted FEV_1_ (p_inter_ = 0.020) and % FEV_1_/FVC (p_inter_ = 0.0033) in the unadjusted analysis, which remained significant after adjusting for covariates (Fig. 1). Individuals with low % predicted FEV_1_ or % FEV_1_/FVC and > median sCD14 levels were more likely to be ever/never smokers with COPD compared to those without COPD.

In a stratified analysis of COPD status and smoking status, we found an inverse relationship between sCD14 levels and lung function among those with COPD (Table 3). Among the ever smokers with COPD, there were statistically significant associations with % predicted FEV_1_ (p_adj_ = 0.0008) and % FEV_1_/FVC (p_adj_ = 0.0002). We found a similar trend for never smokers with COPD; however, our sample size was too small to make statistical inferences. No association between sCD14 levels and lung function measures among those without COPD was observed (Table 3). Similar results were observed when stratifying by COPD status (yes/no) (Additional file 1: Table S1) or the following three categories: COPD; no COPD, current smoker; no COPD, never or former smoker (Additional file 1: Table S2).

**Table 3:** Association of Soluble CD14 Levels with FEV_1_ & FEV_1_/FVC: Stratified by COPD and Smoking Status

*FEV*_*1*_ *(% predicted)*								
	COPD, Ever Smoker		COPD, Never smoker		No COPD, Ever Smoker		No COPD, Never Smoker	
sCD14	n	Mean ± SE	n	Mean ± SE	n	Mean ± SE	n	Mean ± SE
≤ Median	104	69.8 ± 1.7	7	91.2 ± 6.7	121	88.2 ± 1.6	57	89.1 ± 2.3
> Median	104	60.9 ± 1.7	12	78.0 ± 5.1	134	87.9 ± 1.5	37	90.6 ± 2.9
	p	0.0003	p	0.12	p	0.88	p	0.69
	^a^p_adj_	0.0008	^a^p_adj_	0.26	^a^p_adj_	0.91	^a^p_adj_	0.81
*% FEV*_*1*_ */FVC*								
sCD14	n	Mean ± SE	n	Mean ± SE	n	Mean ± SE	n	Mean ± SE
≤ Median	104	61.0 ± 0.74	7	67.0 ± 2.9	121	76.3 ± 0.7	57	77.6 ± 1.0
> Median	104	56.0 ± 0.74	12	62.9 ± 2.2	134	76.3 ± 0.7	37	77.0 ± 1.2
	p	<0.0001	p	0.26	p	0.93	p	0.73
	^a^p_adj_	0.0002	^a^p_adj_	0.53	^a^p_adj_	0.52	^a^p_adj_	0.86

*Abbreviations and Definitions:* sCD14, soluble CD14; FEV_1_, forced expiratory volume in 1 s; % FEV_1_/FVC, FEV_1_/FVC x 100; FVC, forced vital capacity; COPD, FEV_1_/FVC < 0.70; ever smokers, current and former smokers

^a^Multivariable results (p_adj_) are adjusted for age, body mass index, education, sex, race and years worked on a farm

*P*-values adjusted for multiple comparisons with Tukey’s method

### Association of CD14 polymorphisms and haplotypes with sCD14 levels

The study population was genotyped for four polymorphisms at locations -2838, -1720, -651, and -260 relative to the translation start site. The observed minor allele frequencies for the polymorphisms (*CD14*/-2838, A = 25.7%; *CD14*/-651, T = 22.9%; *CD14*/-260, A = 49.4%; CD14/-1720, C =42.0%) showed no deviation from a population in Hardy-Weinberg equilibrium (*p* > 0.05) and were consistent with frequencies reported from the HapMap Project (http:/www.ncbi.nlm.nih.gov/SNP/index.html) in individuals with Northern and Western European ancestry (data not shown). Call rate for all polymorphisms was greater than 95%. The pairwise LD between the four polymorphisms was r2 < 0.8 (Additional file 2: Figure S1). Five haplotypes were identified in the population, and their estimated frequencies were 40.6% for GCCA (-2838, -1720, -651, -260), 26.9% for ATCG, 23.4% for GTTG, 7.2% for GTCA and 1.8% for other. We did not find an association between *CD14* polymorphisms (*CD14*/-2838, *p* = 0.47; *CD14*/-1720, *p* = 0.53*; CD14*/-651, *p* = 0.46; *CD14*/-260, *p* = 0.96;) or haplotypes (*p* = 0.31) with sCD14 levels (≤ median or > median) in the univariate or multivariable analysis (*n* = 584; data not shown).

### Association of CD14 polymorphisms and haplotypes with lung function

We found no significant association between *CD14* polymorphisms and lung function (Table 4). In contrast, we found that the haplotype GTCA was significantly associated with % FEV_1_/FVC even after adjustment for covariates (p_adj_ = 0.021). Individuals with the GTCA haplotype had lower FEV_1_/FVC than those with the GCCA haplotype. There was no association of *CD14* haplotypes with % predicted FEV_1_ (Table 4). We did not find a significant interaction between the COPDsmoke x *CD14* haplotypes on lung function (data not shown).

**Table 4:** Association of *CD14* Polymorphisms and Haplotypes with FEV_1_ & FEV_1_/FVC

*CD14* Genotype	n	FEV_1_ (% Predicted) Mean ± SD	p	^a^p_adj_	% FEV_1_/FVC Mean ± SD	p	^a^p_adj_
rs2569193 *CD14*/-2838							
AA	35	78.3 ± 18.4	0.85	0.98	69.4 ± 10.3	0.97	0.64
AG	228	79.7 ± 21.5			69.5 ± 12.3		
GG	316	80.3 ± 21.4			69.7 ± 11.4		
rs2915863 *CD14*/-1720							
CC	90	80.7 ± 21.7	0.73	0.65	70.3 ± 10.0	0.20	0.13
CT	293	80.3 ± 20.7			69.8 ± 11.8		
TT	180	78.9 ± 21.9			68.6 ± 12.2		
rs5744455 *CD14*/-651							
TT	33	85.3 ± 19.5	0.12	0.071	72.9 ± 8.3	0.14	0.12
TC	193	77.8 ± 21.4			68.6 ± 12.5		
CC	339	80.4 ± 21.3			69.7 ± 11.6		
rs2569190 *CD14*/-260							
AA	134	79.7 ± 21.2	0.71	0.97	70.1 ± 11.4	0.48	0.43
GA	287	79.2 ± 21.7			69.1 ± 12.2		
GG	141	81.0 ± 20.2			70.4 ± 11.0		
Haplotype^b^							
GCCA	245^c^	81.2 ± 1.7	Ref.^d^	Ref.	71.0 ± 1.0	Ref.	Ref
ATCG	149	79.9 ± 1.3	0.41	0.62	70.1 ± 0.7	0.28	0.20
GTTG	134	80.7 ± 1.4	0.76	0.37	70.6 ± 0.7	0.61	0.38
GTCA	44	76.4 ± 2.3	0.056	0.25	66.4 ± 1.3	0.001	0.021
Other	12	90.5 ± 3.7	0.014	0.035	71.3 ± 2.1	0.89	0.81

*Abbreviations and Definitions: FEV*_*1*_ forced expiratory volume in 1 s, % FEV_1_/FVC FEV_1_/FVC x 100; FVC, forced vital capacity

^a^Multivariable results (p_adj_) are adjusted for age, BMI, education, sex, COPD status, race and years worked on a farm

^b^Haplotypes defined as -2838/-1720/-651/-260; Mean ± SE

^c^Sample sizes estimated from haplotype frequency. Mean ± SE are shown

^d^Pairwise comparison to the Base haplotype

Analyses were then conducted to examine the sCD14 x haplotype interaction in relation to % predicted FEV_1_ or % FEV_1_/FVC (Table 5). There was a significant interaction between the GTTG haplotype and sCD14 for % predicted FEV_1_. Individuals with this haplotype and sCD14 levels > median, have on average, 6.94 lower % predicted FEV_1_ values than individuals who possess the GCCA haplotype and sCD14 levels ≤ median (p_inter_ = 0.015). Evidence of interaction was also observed for the GTCA haplotype, though of borderline significance (p_inter_ = 0.061). Individuals with the GTCA haplotype and > median levels of sCD14 have increased FEV_1_/FVC values than those with the GTCA haplotype and ≤ sCD14 levels.

**Table 5:** Interaction of sCD14 and CD14 haplotypes with lung function

Variable	FEV_1_Base model (*n* = 549)	FEV_1_Plus haplotypes (*n* = 545)	FEV_1_/FVC Base model (*n* = 549)	FEV_1_/FVC Plus haplotypes (*n* = 545)
Age, yrs	-0.09 (0.10)	-0.09 (0.10)	-0.07 (0.04)	-0.08 (0.04)
Body mass index, kg/m^3^	-0.37 (0.12)*	-0.34 (0.12)*	0.14 (0.05)*	0.14 (0.05)*
Sex				
Male	Referent	Referent	Referent	Referent
Female	6.17 (5.11)	6.79 (5.14)	4.39 (2.17)*	4.55 (2.18)*
Education				
≤ High School	Referent	Referent	Referent	Referent
> High School	2.51 (1.61)	2.68 (1.62)	1.22 (0.68)	1.12 (0.69)
Race				
White	Referent	Referent	Referent	Referent
Other	2.97 (3.89)	-2.52 (4.42)	0.78 (1.65)	0.23 (1.89)
Worked on Farm, yrs	0.06 (0.04)	0.06 (0.04)	0.01 (0.02)	0.01 (0.02)
COPDSmoke				
No COPD, Never smokers	Referent	Referent	Referent	Referent
No COPD, Ever smokers	0.01 (2.24)	-0.33 (2.25)	0.74 (0.95)	-0.84 (0.96)
COPD	-22.03 (2.3)*	-22.03 (2.31)*	-17.13 (0.98)*	-17.12 (0.98)*
sCD14				
≤ Median	Referent	Referent	Referent	Referent
> Median	-3.19 (1.57)*	0.51 (3.10)	-1.22 (0.66)#	-1.66 (1.31)
Haplotypes				
GCCA		Referent		Referent
ATCG	-	-0.52 (2.00)	-	-1.32 (0.83)
GTTG		1.81 (1.89)		-0.02 (0.80)
GTCA		-2.80 (3.32)		-4.16 (1.40)*
Other		12.53 (4.78)*		1.73 (2.08)
Interaction				
GCCA x ≤ Median sCD14 (n = 111)		Referent		Referent
ATCG x > Median sCD14 (n = 72)	-	-0.11 (2.83)	-	1.39 (1.20)
GTTG x > Median sCD14 (n = 59)		-6.94 (2.85)*		-1.08 (1.21)
GTCA x > Median sCD14 (n = 24		0.05 (4.45)		3.54 (1.89)#
Other x > Median sCD14 (n = 6)		-9.54 (6.46)		-3.78 (2.78)

*Abbreviations and Definitions:* FEV_1_, forced expiratory volume in 1 s; % FEV_1_/FVC, FEV_1_/FVC x 100; FVC, forced vital capacity

^Haplotypes defined as -2838/-1720/-651/-260

*p < 0.05, #p < 0.07

## Discussion

CD14 is an acute phase protein involved in LPS signaling and therefore is essential for interfacing the innate immune system with PAMPs. Studies have found that airway inflammation and decreased pulmonary function are common among farmers, and these findings are linked to the presence of LPS in inhaled organic dust [12]. The importance of CD14 in inflammatory processes is underscored by its association with a multitude of diseases, including sepsis, cardiovascular disease, periodontitis, tuberculosis and atopic asthma [29–33]. CD14 represents an important mediator of lung function, as we have shown previously that *CD14* haplotypes (*CD14*/-1720G or *CD14*/-260A) are associated with decreased lung function among those exposed to agricultural environments [34]. The current study presents new evidence for the relationship between sCD14 levels and pulmonary function among agricultural workers and haplotype x sCD14 interaction with lung function.

Specifically we found that sCD14 levels were inversely related to % predicted FEV_1_ and FEV_1_/FVC; however, this association was found only in COPD patients that were ever smokers compared to those without COPD (ever/never smokers). Though there have been no studies investigating the association between sCD14 levels and lung function, in a case-control study of nine never smokers, 10 healthy smokers and 10 COPD patients, Reguiro et al. showed that sCD14 levels in the bronchoalveolar lavage fluid were elevated among healthy smokers and patients with COPD compared to never-smokers [18]. We found a similar but non statistically significant relationship in our agriculturally-exposed population. This discrepancy is most likely due to measurement of sCD14 levels in two different compartments, the bronchoalveolar lavage fluid of the lung and blood serum.

The reasons for the association between sCD14 concentration and reduced pulmonary function among those with COPD are not fully understood, but several mechanisms may be involved. Reduced lung function among COPD patients may be responsible, in part, for the observed systemic inflammation as measured by sCD14. Inflammatory lung or pulmonary epithelial cells have been shown to express IL-6. IL-6 may reach the liver via the bloodstream, stimulating the acute phase response and production of sCD14 by the liver and sequentially activating pulmonary inflammatory cells during transit through the pulmonary circulation. An alternative mechanism -reverse causation- cannot be excluded: high levels of cytokines and acute phase reactants in the peripheral circulation as a consequence of cigarette smoking may be a cause rather than a consequence of poor lung function. Persistence of elevated sCD14 concentration and systemic inflammation may result in damage to the airways, and lower FEV_1_ of COPD patients.

The marginal associations between *CD14* genotypes and sCD14 concentrations may not be surprising, given the equivocal results reported to date. While some studies have shown significant association of the same tagging polymorphisms examined in our study with sCD14 concentration, others failed to show any associations [14, 19, 20, 35–38]. These inconsistencies may be driven by the complex gene-environment interactions of CD14 expression as well as the use of relatively healthy versus diseased subjects where any genetic influences on sCD14 are simply overshadowed by other inflammatory stimuli. The masking of the polymorphism-protein signal via inflammation was convincingly reported by measuring sCD14 levels pre- and post- endotoxin inhalation; *CD14*/-260 and *CD14*/-1720 were associated with sCD14 levels pre-inhalation yet not post-inhalation [14]. Recently, it has been shown that the association between *CD14* polymorphisms and sCD14 levels decreases from birth to 10 years of age and that this lack of association is paralleled with a significant increase in *CD14* methylation. Importantly, *CD14* methylation levels were inversely associated with sCD14 levels. Further studies are necessary to understand the impact of agricultural exposure and *CD14* methylation on sCD14 levels in this population [38].

There have been a limited number of studies in adults showing an association between *CD14* polymorphisms and several lung phenotypes such as asthma and allergy [39, 40], wheeze [41], pulmonary tuberculosis [42] and pulmonary function [41, 43–45]. While the results of the present study show a weak association between *CD14* polymorphisms and lung function, we found a significant interaction between *CD14* haplotypes and sCD14 levels on lung function. Many multiplicative interactions have been found with *CD14* polymorphisms and environmental exposures. In a study of Dutch farmers and agriculture industry workers, increased environmental exposure to endotoxin in those with the *CD14*/-1720 T or *CD14*/-260G allele was associated with lower FEV_1_ values compared to those homozygous for the C or A allele [41]. In contrast, in a study of laboratory animal workers, those with high endotoxin exposure and the *CD14*/-1720C allele had significantly lower lung function than workers with the TT genotype [43]. In another study of adults, pack-years of smoking was found to modify the association between *CD14*/-260 and lung function (FEV_1_ or FEV_1_/FVC) [45]. In this study, the *CD14*/-260AA genotype was associated with lower lung function in moderate smokers, while heavy smokers with the CC genotype had decreased lung function [45]. These disparate results underscore the complex nature of the CD14 gene and the importance of integrating genetic, epigenetic and environmental exposures for understanding determinants of the outcome.

A strength of this study is that rather than selecting only polymorphisms with known biologic function, our approach involved the use of tagging polymorphisms with the broader goal of capturing the overall polymorphic nature of the gene. Therefore it is possible that the haplotypes examined in this study and shown to interact with sCD14 may have little functional biologic consequence. For instance, the haplotype GTTG was shown to interact with serum sCD14 levels. Each polymorphism in this haplotype is found in the promoter region of the *CD14* gene, yet only one locus has been shown to be functional, *CD14*/-260 [36]. It is possible that the *CD14*/-2838, *CD14*/-1720 and *CD14*/-651 are not functional but are merely in LD with other regions of the gene. The *CD14*/-260 polymorphism has been shown to be functional via modifying the transcriptional activity via SP transcription factors, with the A allele having more gene expression than the G allele [36].

To our knowledge, this is the first study to show an association between sCD14 levels, *CD14* haplotypes and lung function in an occupationally-exposed population. However the question remains as to whether agricultural exposure plays a role in our findings. Replication of this study in participants without agricultural exposure would be necessary to address this issue. A limitation of using an exposed population is that a “healthy worker effect” may have resulted in bias towards the null, because individuals with respiratory problems may avoid agricultural jobs with the highest exposures. Due to the cross-sectional nature of this study, we were unable to account for this type of bias. Furthermore, we were unable to measure LPS in the occupational setting to determine whether the association between sCD14 and lung function was modified by LPS exposure intensity, an area that warrants further study. In addition our population was composed of mostly Caucasian males and additional population studies with other ancestries would be important information.

## Conclusions

In summary, we have shown that levels of sCD14 are inversely associated with pulmonary function and are significantly modified by *CD14* haplotypes among those with COPD in this agriculturally-exposed population. Further studies are needed to elucidate the implications and potential effects of higher circulating levels of sCD14 in patients with impaired pulmonary function.

## Additional file

Additional file 1: Interaction of CD14 Haplotypes And Soluble CD14 On Lung Function in Agricultural Workers. (DOC 48 kb)
Additional file 2: Figure S1. Linkage disequilibrium (LD) between 4 SNPs analyzed for CD14. LD values presented as r2x100. (TIF 44 kb)
