# Supplementary material for: Interaction of CD14 haplotypes and soluble CD14 on pulmonary function in agricultural workers
Source: Respir Res. 2017 Mar 16;18:49. doi: 10.1186/s12931-017-0532-y (PMC5353891; doi:10.1186/s12931-017-0532-y)
Supplement: Additional file 1: — Interaction of CD14 Haplotypes And Soluble CD14 On Lung Function in Agricultural Workers. (DOC 48 kb) [file 12931_2017_532_MOESM1_ESM.doc]

**Table S1. Interaction of Soluble CD14 and COPD on FEV_1_ & FEV_1_/FVC**

| ***FEV_1_ (% predicted)***  ^p_adj-inter_ = 0.011 | | | | | | | | |
| --- | --- | --- | --- | --- | --- | --- | --- | --- |
|  | COPD | | | | No COPD | | | |
| sCD14 | n | Mean ± SD | p | *p_adj_ | n | Mean ± SD | p | *p_adj_ |
| ≤ Median | 111 | 71.2 ± 19.3 | 0.0014 | 0.0066 | 181 | 88.3 ± 16.5 | 0.99 | 1.0 |
| > Median | 119 | 62.3 ± 20.8 |  |  | 173 | 88.8 ± 16.8 |  |  |
| ***% FEV_1_/FVC***  ^p_adj-inter_ = 0.0033 | | | | | | | | |
|  | COPD | | | | No COPD | | | |
| sCD14 | n | Mean ± SD | p | *p_adj_ | n | Mean ± SD | p | *p_adj_ |
| ≤ Median | 111 | 61.3 ± 9.5 | <0.0001 | 0.0031 | 181 | 76.7 ± 4.3 | 0.99 | 0.98 |
| > Median | 119 | 56.6 ± 12.3 |  |  | 173 | 76.5 ± 4.4 |  |  |

*Abbreviations and Definitions:* sCD14, soluble CD14; FEV_1_, forced expiratory volume in 1 sec; % FEV_1_/FVC, FEV_1_/FVC x 100; FVC , forced vital capacity; COPD, FEV_1_/FVC < 0.70.

***Multivariable results (p_adj_) are adjusted for age, BMI, education, sex, race and years worked on a farm.

^Multivariable interaction results are adjusted for age, BMI, education, sex, race and years worked on a farm.

**Table S2. Interaction of Soluble CD14 Levels and COPD/Smoking Status on FEV_1_ & FEV_1_/FVC**

| ***FEV_1_ (% predicted)***  ^p_adj-inter_ = 0.043 | | | | | | | | | | | | |
| --- | --- | --- | --- | --- | --- | --- | --- | --- | --- | --- | --- | --- |
|  | COPD | | | | No COPD, Current Smoker | | | | No COPD, Never/Former Smoker | | | |
| sCD14 | n | Mean ± SD | p | *p_adj_ | n | Mean ± SD | p | *p_adj_ | n | Mean ± SD | p | *p_adj_ |
| ≤ Median | 111 | 71.2 ± 19.3 | 0.0032 | 0.043 | 32 | 88.0 ± 14.8 | 0.91 | 0.87 | 146 | 88.6 ± 16.9 | 0.99 | 0.98 |
| > Median | 119 | 62.3 ± 20.8 |  |  | 19 | 82.6 ± 14.2 |  |  | 152 | 89.2 ± 16.8 |  |  |
| ***% FEV_1_/FVC***  ^p_adj-inter_ = 0.015 | | | | | | | | | | | | |
|  | COPD | | | | No COPD, Current Smoker | | | | No COPD, Never/Former Smoker | | | |
| sCD14 | n | Mean ± SD | p | *p_adj_ | n | Mean ± SD | p | *p_adj_ | n | Mean ± SD | p | *p_adj_ |
| ≤ Median | 111 | 61.3 ± 9.5 | <0.0001 | 0.0030 | 32 | 75.0 ± 4.2 | 0.99 | 0.99 | 146 | 77.0 ± 4.3 | 0.99 | 0.99 |
| > Median | 119 | 56.6 ± 12.3 |  |  | 19 | 76.4 ± 2.9 |  |  | 152 | 76.5 ± 4.5 |  |  |

*Abbreviations and Definitions:* sCD14, soluble CD14; FEV_1_, forced expiratory volume in 1 sec; % FEV_1_/FVC, FEV_1_/FVC x 100; FVC, forced vital capacity; COPD, FEV_1_/FVC < 0.70.

*Multivariable results (p_adj_) are adjusted for age, body mass index, education, sex, race and years worked on a farm.

^Multivariable interaction (p_inter_) results are adjusted for age, body mass index, education, sex, race and years worked on a farm.
