# Supplementary figures and images for: Interaction of CD14 haplotypes and soluble CD14 on pulmonary function in agricultural workers
Source: Respir Res. 2017 Mar 16;18:49. doi: 10.1186/s12931-017-0532-y (PMC5353891; doi:10.1186/s12931-017-0532-y)

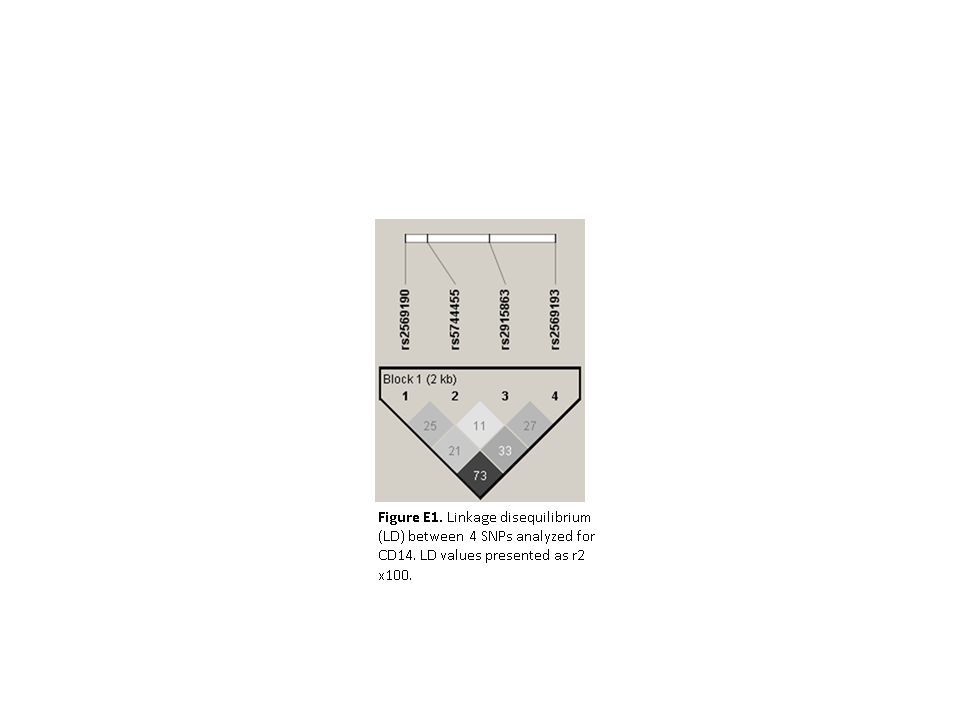

Supplement: Additional file 2: Figure S1. — Linkage disequilibrium (LD) between 4 SNPs analyzed for CD14. LD values presented as r2x100. (TIF 44 kb) [file 12931_2017_532_MOESM2_ESM.tif]
